# Seed Location Impacts Whole-Brain Structural Network Comparisons between Healthy Elderly and Individuals with Alzheimer’s Disease

**DOI:** 10.3390/brainsci7040037

**Published:** 2017-04-06

**Authors:** Lauren Zajac, Bang-Bon Koo, Corinna M. Bauer, Ron Killiany, Behalf of the Alzheimer’s Disease Neuroimaging Initiative

**Affiliations:** 1 Department of Anatomy & Neurobiology, Boston University School of Medicine, Boston, MA 02118, USA; bbkoo@bu.edu (B.K.); killiany@bu.edu (R.K.); 2 Center for Biomedical Imaging, Boston University School of Medicine, Boston, MA 02118, USA; 3 Department of Ophthalmology, Massachusetts Eye and Ear Infirmary, Harvard Medical School, Boston, MA 02114, USA; Corinna_Bauer@meei.harvard.edu

**Keywords:** DTI, Alzheimer’s disease, graph theory, structural networks, MRI, diffusion

## Abstract

Whole-brain networks derived from diffusion tensor imaging (DTI) data require the identification of seed and target regions of interest (ROIs) to assess connectivity patterns. This study investigated how initiating tracts from gray matter (GM) or white matter (WM) seed ROIs impacts (1) structural networks constructed from DTI data from healthy elderly (control) and individuals with Alzheimer’s disease (AD) and (2) between-group comparisons using these networks. DTI datasets were obtained from the Alzheimer’s disease Neuroimaging Initiative database. Deterministic tractography was used to build two whole-brain networks for each subject; one in which tracts were initiated from WM ROIs and another in which they were initiated from GM ROIs. With respect to the first goal, in both groups, WM-seeded networks had approximately 400 more connections and stronger connections (as measured by number of streamlines per connection) than GM-seeded networks, but shared 94% of the connections found in the GM-seed networks. With respect to the second goal, between-group comparisons revealed a stronger subnetwork (as measured by number of streamlines per connection) in controls compared to AD using both WM-seeded and GM-seeded networks. The comparison using WM-seeded networks produced a larger (i.e., a greater number of connections) and more significant subnetwork in controls versus AD. Global, local, and nodal efficiency were greater in controls compared to AD, and between-group comparisons of these measures using WM-seeded networks had larger effect sizes than those using GM-seeded networks. These findings affirm that seed location significantly affects the ability to detect between-group differences in structural networks.

## 1. Introduction

Diffusion magnetic resonance imaging (MRI) enables white matter structure to be studied in vivo. Methods such as diffusion tensor imaging (DTI) have led to the ability to quantify the amount of diffusion anisotropy in individual voxels in the brain as a surrogate of white matter integrity [1]. Alternatively, tractography has been used to virtually dissect white matter tracts and construct whole-brain networks [2,3,4,5,6,7]. Network analysis methodologies, such as graph theory, have been applied to the whole-brain networks built using diffusion tractography. In these analyses, whole-brain networks are modeled as a graph comprised of a set of nodes, which are typically cortical or subcortical regions, and edges, which are the connections between them. 

To construct a whole-brain structural network from DTI data, a number of methodological choices are involved from image acquisition through tractography. The effect of different parameters on network construction is gaining attention ([8,9,10,11,12,13], see [14] for review) because there is currently no universally-accepted standard set of parameters. As a result, most published studies use different methods to construct networks from diffusion MRI data. One important choice in constructing a whole-brain structural network is how to define the nodes of the network (see [14,15] for review). The number of nodes, how they are defined (e.g., which atlas, if any, is used), and how they are used in the construction of the network have been shown to affect network properties such as small-worldness [8] and hub identity [9]. While some effects of node choice have been assessed within the same subjects, the influence of the tissue in which the node is placed (e.g., gray or white matter) has not been evaluated in the context of disease.

One such disorder in which structural network analysis may be influenced by the location of nodes is Alzheimer’s disease (AD). AD is the most prevalent form of dementia and has been characterized as a “disconnection syndrome” [16,17]. Neurofibrillary tangles preferentially accumulate in pyramidal neurons in association cortices in layers 2/3 and 5, the neurons of which make long cortico-cortical connections [18,19,20]. Furthermore, amyloid beta accumulates within the highly-connected hubs of the default mode network, which are also comprised of association cortex [21]. The accumulation of pathology in brain hubs and the progressive degeneration of the neurons whose axons form long-range cortico-cortical connections inevitably affect the integrity and microstructure of the white matter. This pattern of AD neuropathology supports the characterization of AD as a disconnection syndrome and makes it suitable for study through the analysis of whole-brain structural networks built from diffusion MRI data.

A considerable amount of research has reported significant changes associated with AD in gray matter [22,23,24,25,26], as well as white matter [27,28,29,30,31,32,33]. Furthermore, tractography has been utilized to examine how connectivity between specific regions of interest (ROIs) [34,35,36] or within whole-brain structural networks [37,38,39,40,41,42] differs between healthy aging and individuals with AD. These studies have all shown decreased connectivity in AD relative to controls; however, specific differences between controls and AD in these studies vary. Importantly, the whole-brain structural network studies that have been conducted used different network construction methods and connection weighting, making a direct comparison of the results difficult [14]. This speaks to the need for a common methodology in network construction, if plausible. Regardless, a better understanding of the effects of network parameters on constructed networks and between-group comparisons that use them is needed.

The current analysis focuses on the intersection between DTI network construction and between-group differences—focusing on how node location affects whole-brain structural networks constructed in healthy elderly controls and individuals with AD. To this end, we constructed two networks for each subject using a seed-to-target ROI-based strategy and deterministic tractography. In one network, seeding was initiated in white matter ROIs (WM-seed networks), and in the second network, seeding was initiated in gray matter ROIs (GM-seed networks). GM ROIs were utilized as targets in both networks. Tissue elements (i.e., axons, dendrites, blood vessels, cell bodies) in GM voxels have mixed orientations, especially at the scale of ~2–3 mm. Despite this, a large number of GM voxels still survive the FA threshold, are included in the tractography algorithm, and can thus be used to generate brain networks with a large number of connections that satisfy small world criteria. GM voxels are in close proximity to both the surface of the brain, adjacent to cerebrospinal fluid (CSF), and white matter. In CSF voxels, there are no major barriers to the diffusion of water molecules, and in WM voxels, there are strong and oriented barriers to the diffusion of water molecules. Within a given GM voxel, there may be partial voluming with CSF and/or WM depending on where that voxel is located, leading the direction of its primary eigenvector to be significantly different from that of the primary eigenvectors of the GM or WM voxels that abut it. In contrast, WM voxels primarily contain axons in compacted and ordered structure. Though their orientation is far from completely uniform within a voxel at the scale of ~2–3 mm, the fractional anisotropy (FA) of WM voxels tends to be higher than GM voxels due to the overall geometry of axon packing. Additionally, WM voxels are more likely to have primary eigenvectors whose direction is more similar to the WM voxels that abut it, thus making the continuation of streamline tracking more likely. Both seeds located on the gray matter-white matter boundary [9,43,44,45] and GM seeds [46,47,48,49] have been used with seed-to-target ROI-based strategies to generate structural brain networks. Yet, to our knowledge, no systematic evaluation of the effects of seed type on network comparisons has been performed. These factors motivated the following analyses and led us to predict that WM-seed networks would contain a greater number of streamlines between ROIs (i.e., “stronger” connections) than GM-seed networks and that this would change or enhance differences in network measures found between groups.

## 2. Materials and Methods

### 2.1. Subjects

The data used in this study were obtained from the Alzheimer’s Disease Neuroimaging Initiative (ADNI) database [50]. The ADNI started in 2003 and its primary goal has been to test whether the development of mild cognitive impairment (MCI) and AD can be measured using a combination of imaging techniques, biomarkers, and neuropsychological assessment. For current information on the ADNI, see [51].

Ten healthy elderly individuals (5 female) and ten individuals with AD (5 female) who underwent DTI scanning were selected from the Alzheimer’s disease Neuroimaging Initiative (ADNI) database. The ADNI is a multisite study, and ADNI participants are diagnosed at their respective sites using the global Clinical Dementia Rating (CDR) scale and neuropsychological test scores. Diagnoses are confirmed by the ADNI Clinical Core and reassessed once a year. Subject ID numbers, ages, and global CDR scale scores of the individuals whose data were used in this study are listed in Table S1. In planning for this study it was important to balance two factors. The first was to limit the number of subjects included in order to ensure that any significant differences found could only come from large effects. This provides protection from finding statistically significant but meaningless differences. The second was to use subject selection criteria that would prevent bias in subject selection that could influence the findings. To this end, we used a pseudo-random selection method to select the subjects for the AD and control groups, starting from the lowest site number at which DTI scans were acquired. Sex was intentionally balanced within and between groups. Control subjects had an average age of 69.5 (±4.9) and AD subjects had an average age of 70.1 (±10.2). A two-sample, two-tailed *t*-test with unequal variance showed no significant differences in the age of subjects in each group (*p* = 0.865, *d* = 0.08). All control subjects had a global CDR of 0. Eight AD subjects had a CDR of 1 and the remaining two AD subjects had a CDR of 2. All subjects gave informed consent at the institutions where data were collected upon enrollment in the ADNI.

### 2.2. Imaging Data

T1-weighted structural scans and DTI datasets were downloaded from the ADNI database in their original DICOM (Digital Imaging and Communications in Medicine) format. Scans used in this study were acquired on 3T GE scanners at multiple scanning sites. DTI data contained five volumes acquired with a *b*-value of 0 and 41 directions acquired with a *b*-value of 1000. Additional scan parameters can be found on the LONI website [50]. All scans were converted to NIfTI (Neuroimaging Informatics Technology Initiative) format using dcm2nii and visually inspected prior to use.

The DTI data used in this study was acquired at a resolution of 2.73 × 2.73 × 2.7 mm and reconstructed as 1.37 × 1.37 × 2.7 mm, which is the resolution of the data in the ADNI database. After converting to NIfTI, FLIRT v6.0 [52,53] in FSL v5.0.8 was used to reslice each DTI dataset to its original, nearly isotropic resolution of 2.73 × 2.73 × 2.7 mm prior to further processing.

### 2.3. Network Construction

A brain network can be modeled as a graph, which is a set of nodes and edges ([54,55], see [56] for review). In this study, we represented structural brain networks as weighted, undirected graphs. The nodes of our structural networks were either gray matter or white matter ROIs and the edges were streamlines generated between pairs of ROIs using deterministic tractography. Edge weights were equal to the number of streamlines generated between each pair of ROIs with large and small numbers of streamlines representing strong and weak connections, respectively.

#### 2.3.1. Parcellation of Gray Matter and White Matter

Cortical surfaces were generated from each subject’s T1-weighted scan in FreeSurfer v5.3.0 [57] (Laboratory for Computational Neuroimaging, Athinoula A. Martinos Center for Biomedical Imaging, Charlestown, MA, USA) using the standard pipeline. Once the surfaces were created, errors in each subject’s pial and white matter surfaces were edited manually. A total of 68 cortical gray matter (GM) ROIs were generated using the Desikan-Killiany atlas [58] (34 per hemisphere) and used as nodes in the whole-brain network. A total of 8 subcortical ROIs (4 per hemisphere) were generated and used in the whole-brain network as well: amygdala, hippocampus, putamen, and thalamus. Each subject’s GM ROIs were generated in T1 space and transformed into DTI space using bbregister, a FreeSurfer tool that uses a boundary-based cost function for image registration [59]. This tool performs within-subject, cross-modal coregistration of images through use of the gray matter-white matter boundary generated from a subject’s T1-weighted anatomical image. This coregistration was used to transform ROIs in T1 space into DTI space. A selection of GM ROIs were overlaid onto each subject’s DTI data and visually inspected prior to fiber tracking to ensure accurate transformation into DTI space.

**Figure 1:**
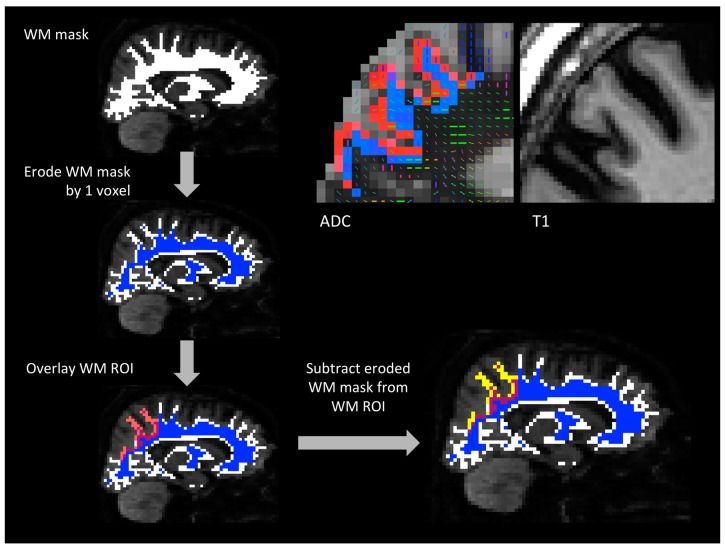
Generating WM ROIs. (**top left**) one subject’s white matter mask overlaid on that subject’s diffusion-weighted MRI data. The WM mask was eroded by one voxel and the resulting mask is shown in blue. Then, WM ROIs generated in FreeSurfer and transformed into diffusion space (one example shown in transparent red) were overlaid on the eroded WM mask. The eroded WM mask was subtracted from each WM ROI to isolate the strip of voxels just deep to the GM/WM interface that was used as the final WM ROI (shown in yellow). (**top right**) one subject’s left superior parietal cortex GM ROI (red) and WM ROI (blue) overlaid on an apparent diffusion coefficient (ADC) map. The corresponding region on that subject’s T1-weighted image is shown on the far right. GM and WM ROIs were transformed from T1 space to DTI space using bbregister, which relies on the gray matter-white matter boundary for image alignment. The bright voxels lining the GM ROI are CSF and the dark voxels deep to the WM ROI are white matter. On the left, the primary diffusion direction of each voxel is visible and the map is thresholded at FA = 0.1. In this section, a greater number of WM voxels survive the FA threshold compared to the voxels in the GM ROI (voxels that survive the FA threshold have a diffusion tensor in them). CSF: cerebrospinal fluid, DTI: diffusion tensor imaging, FA: fractional anisotropy, GM: gray matter, MRI: magnetic resonance imaging, ROIs: regions of interest, WM: white matter.

White matter (WM) ROIs were one-voxel thick and lined the white matter surface just deep to the gray matter ROIs (Figure 1). To create these WM ROIs, whole-brain cerebral white matter volumes generated in FreeSurfer were eroded by one voxel and this mask was subtracted from each white matter ROI derived from the standard FreeSurfer white matter parcellation [31]. The FreeSurfer white matter parcellation divides the entire cerebral white matter volume into 68 regions that correspond to the 68 GM regions produced in the cortical parcellation. Subtraction of the eroded cerebral white matter volume from each white matter ROI allowed the one-voxel strip of white matter just under the gray matter-white matter boundary corresponding to that ROI to be isolated. One-voxel-thick WM ROIs were chosen to minimize the seeding volume while maintaining anatomical relevance to the GM ROIs. WM ROIs were generated in T1 space and transformed into DTI space using the same methods used to transform GM ROIs. A selection of WM ROIs were overlaid onto each subject’s DTI data and visually inspected prior to fiber tracking.

The same subcortical ROIs were used to generate WM-seed and GM-seed networks because they contain a mixture of white matter and gray matter that cannot be resolved at the spatial resolution of this MRI dataset.

#### 2.3.2. Tractography

Two structural networks were generated for each subject using a seed-to-target ROI-based method. In evaluating the connections between region A and region B using a seed-to-target strategy, fiber tracking is initiated in region A *n* times. The number of streamlines that reach region B corresponds to the strength of the connection between regions A and B. To evaluate the connections between region A and regions C, D, E, etc., this process is repeated for each ROI pair. One network was generated using GM ROIs as seed regions and the second network was generated using the WM ROIs as seed regions. These networks will be referred to as GM-seed and WM-seed networks, respectively, throughout the rest of the paper. In both instances, target ROIs were placed in the gray matter.

Deterministic fiber tracking was used to generate whole-brain structural networks from DTI data using an in-house MATLAB script employing DSI Studio (24 June 2013 build) functions [60]. The DTI data were reconstructed and a tensor model fit to each voxel using the DTI reconstruction scheme implemented within DSI Studio to determine the three eigenvalues and eigenvectors for each voxel [61]. The principle eigenvector of each voxel was used in fiber tracking. For each pair of seed and target ROIs, 10,000 seeds to initiate fiber tracking were randomly placed within voxels that survived the FA threshold in the seed ROI. Fiber tracking continued in both directions until a voxel with an FA lower than 0.1 or an angle exceeding 45° between steps was encountered. Only streamlines that reached the target ROI were counted and entered into the connectivity matrix. The following additional fiber tracking parameters were used: 2 mm step size, smoothing of 0.5, minimum fiber length of 5 mm, maximum fiber length of 300 mm, random subvoxel seeding, Gaussian radial interpolation, and the RK4 tractography algorithm. Additional corrections that removed looping streamlines were also carried out.

Prior to analysis, networks were symmetrized because DTI data does not contain directional information. Any asymmetry in connection strength (i.e., number of streamlines) present after fiber tracking for a given seed-target ROI pair is the result of ROI volume, differences between the shape and location of seed and target regions, differences in seed placement, and accumulated error as streamlines are generated over long distances. To symmetrize the matrices, the higher number of streamlines between each pair of ROIs was used as the weight of each connection. This sequence of processing steps resulted in whole-brain networks in which each connection was weighted by the number of streamlines generated between ROIs. 

### 2.4. Graph-Theory Network Measures

#### Whole-Network Measures

In the past several years, graph theory has been applied to the study of human brain networks (see [56] for review). Small-worldness [62] is one of the most common topological measures examined within human brain networks. Both binary and weighted structural and functional human brain networks have been characterized as having small-world properties [63]. Thus, small-worldness of WM-seed and GM-seed networks was examined in both controls and AD to ensure that networks generated using both types of seed ROIs satisfied small-world criteria. 

Because weighted networks were used in this study, small-worldness was evaluated using global and local efficiency rather than path length or clustering coefficient [64,65]. Note, that global and local efficiency are similar to path length and clustering coefficient, respectively, which are validated to define small-worldness in binary networks [62,64,65]. Global efficiency was defined as:

(1) $$E_{\mathrm{glob}}=\frac{1}{n}\sum_{i\in N}\frac{\sum_{j\in N,j\neq i}{\left(d_{ij}^{w}\right)}^{-1}}{n-1},$$

where *n* is the number of nodes and $d_{ij}^{w}$ is the shortest weighted path length between nodes *i* and *j*. The local efficiency was defined as:

(2) $$E_{\mathrm{loc}}=\frac{1}{n}\sum_{i\in N}\frac{\sum_{j,h\in N,j\neq i}(w_{ij}w_{ih}[d_{jh}^{w}{\left(N_{i}){]}^{-1}\right)}^{1/3}}{k_{i}\left(k_{i}-1\right)},$$

where *n* is the number of nodes, *w_ij_* and *w_ih_* are the weights of the connections between nodes *i* and *j* and nodes *i* and *h*, respectively, *k_i_* is the degree of node *i*, and $d_{jh}^{w}\left(N_{i}\right)$ is the shortest weighted path length between nodes *j* and *h* that only contains neighbors of *i*. Efficiency measures were computed using Brain Connectivity Toolbox (BCT, Version 2015-25-01) functions [66].

For all WM-seed and GM-seed networks, global efficiency and local efficiency were calculated. A small-world network was defined as having global efficiency slightly lower than that of a random network and local efficiency higher than that of a random network [64,65]. For each subject, the average global and local efficiencies of 100 random weighted networks were calculated to evaluate small-worldness. Each random network was generated through 100 rewirings of that subject’s network at each node and preserved the number of nodes and edges, the degree distribution, and the strength distribution of the original network. The average correlation between the strength distributions of each subject’s original network and each of the 100 random networks was higher than 0.9.

Density and average strength of all networks were calculated. Density was defined as:

(3) $$D=\frac{e}{N\left(N-1\right)},$$

where *e* is the number of edges in the network and *N* is the total number of nodes. Density is the ratio of edges in a network to the total number of possible edges. Average network strength was determined by first calculating the strength (i.e., weighted degree) of each node in the network and then averaging the strength of all nodes in the network.

### 2.5. Statistics

Paired *t*-tests were used to determine whether significant within-group differences were present between WM-seed and GM-seed networks for both controls and AD subject groups. Two-sample *t*-tests were utilized to assess differences between controls and AD in either WM-seed networks or GM-seed networks. Equality of variance was tested using F-tests prior to performing two-sample *t*-tests, and all effect sizes are presented as Cohen’s *d* values. Statistics were performed in MATLAB vR2012b (MathWorks, Natick, MA, USA) and JMP Pro v12.1.0 (SAS, Cary, NC, USA).

#### 2.5.1. Network Based Statistic (NBS)

The Network-Based Statistic (NBS) toolbox v1.2 [67] was used to determine whether significantly different networks were present between WM-seed and GM-seed networks in both control and AD groups. In addition, NBS was utilized to identify significantly different networks between controls and AD using both WM and GM seeds. NBS controls for multiple comparisons through cluster-based thresholding, where connected components of a network are considered a cluster [67]. This approach is more sensitive to detecting differences compared to alternative methods of correcting for multiple comparisons. However, one drawback to this technique is that no individual connection in a significant network can be considered significant on its own.

Another widely acknowledged drawback to NBS is the arbitrary nature of threshold choice [67]. We chose our thresholds based on two main criteria: (1) NBS results containing one significant network rather than several and (2) *p*-value of the significant network. For our within-group comparisons of WM-seed to GM-seed networks for both controls and AD, an NBS threshold of 3.2 was used. For our between-group comparisons of WM-seed networks and GM-seed networks in controls and AD, an NBS threshold of 2 was used. These thresholds resulted in one significant network with a low *p*-value for all our comparisons that showed significant results. All other NBS parameters were the same for all comparisons: *t*-tests performed at every connection, a *p*-value threshold of 0.05 for every connection, and 5000 permutations. The size of the significant component was measured based on extent, not intensity.

### 2.6. Node-Based Measures

The networks identified using the NBS toolbox (see Section 2.5.1) were used to target nodes for further investigation. Specifically, nodal efficiency (e.g., the average shortest path length between a node and all other nodes in a network [68]) was examined for nodes with a large number of differential connections in the WM-seed and GM-seed NBS networks demonstrating differences between controls and AD. BCT functions were used as part of an in-house script to calculate the nodal efficiency of NBS network-targeted nodes. Nodal efficiency, for node *i*, was defined as:

(4) $$E_{\mathrm{node},i}=\frac{\sum_{j\in N,j\neq i}{\left(d_{ij}^{w}\right)}^{-1}}{n-1},$$

where *n* is the number of nodes and $d_{ij}^{w}$ is the shortest weighted path length between nodes *i* and *j*.

## 3. Results

### 3.1. WM-Seed and GM-Seed Network Characterization and Comparisons

The average number of edges in the WM-seed and GM-seed networks and the average percentage of edges present in both networks are listed in Table 1. Every subject’s WM-seed network contained a greater number of edges than his/her respective GM-seed network. The vast majority of connections in each subject’s GM-seed network were present within each subject’s WM-seed network. Thus, seeding in WM ROIs provides additional connections that are not captured when seeding in GM ROIs. The percentage of GM-seed connections present in WM-seed networks was not significantly different between AD and controls (*p* = 0.77, *d* = 0.16).

Network density of WM-seed networks was significantly greater than GM-seed networks in AD (*p* = 4.99 × 10^−9^) and controls (*p* = 7.89 × 10^−8^) (see Table 2). Network density did not differ significantly between AD and control groups for either WM-seed (*p* = 0.54, d = 0.29) or GM-seed networks (*p* = 0.051, d = 0.91).

The average strength of WM-seed networks was significantly higher than the average strength of GM-seed networks in both AD (*p* = 1.63 × 10^−8^) and controls (*p* = 5.44 × 10^−7^) (see Table 2). Network strength did not differ significantly between AD and control groups for either WM-seeds (*p* = 0.18, d = 0.44) or GM-seeds (*p* = 0.44, d = 0.069).

### 3.2. Within-Group Differences in WM-Seed versus GM-Seed Networks

**Figure 2:**
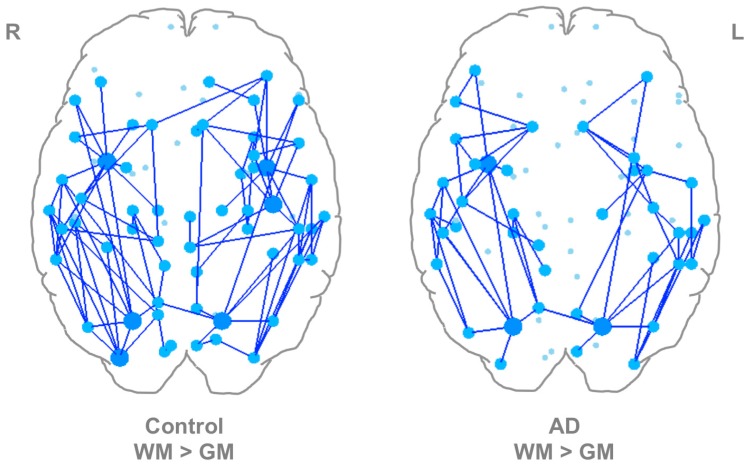
Within-Group Comparisons of WM-Seed and GM-Seed Networks using NBS. (**left**) Control WM > GM. (**right**) AD WM > GM. Large, dark blue nodes have 6 or more differential connections. Medium light blue nodes have between 1 and 5 differential connections. Small light blue nodes were not part of the significant network. AD: Alzheimer’s disease, GM: gray matter, NBS: Network Based Statistic, WM: white matter.

As a next step, matrices created from WM or GM seeds were compared for each subject using NBS to determine if systematic differences in network topology existed based on seed location. This was done separately for control and AD groups. NBS revealed significantly stronger networks using WM-seeds compared to GM-seeds in both controls and AD. In controls, this network was composed of 59 nodes and 95 edges (Figure 2) (*p* < 0.001). In AD, this network was comprised of 41 nodes and 57 edges (Figure 2) (*p* < 0.001). Thirty-eight edges were shared between the significant control and AD subnetworks. The nodes identified with 6 or more differential connections in controls and AD are listed in Table 3 and full network details can be found in Table S2. No significantly stronger networks were found when GM-seed networks were compared to WM-seed networks in either controls or AD.

To further explore the difference between seeding in WM ROIs versus GM ROIs, we examined whether this difference could be explained by FA-thresholded ROI volume. FA-thresholded ROI volume is related to connection strength in the tractography method we used to generate networks in this study because 10,000 seeds were placed in each ROI after FA thresholding, regardless of the number of voxels in each ROI. To illustrate this concept, assume that the streamlines from two voxels in ROI X are responsible for generating the majority of the streamlines between ROI X and ROI Y. If the same number of seeds is randomly placed in ROI X when it contains 10 voxels versus 20 voxels, there is a higher probability of a greater number of streamlines forming between ROI X and ROI Y when ROI X contains 10 voxels compared to 20 voxels. Because a majority of WM ROIs had fewer voxels after FA-thresholding than their respective GM ROIs, we examined whether this could account for the higher average connection strength in WM-seed networks.

To investigate the relationship between seed ROI volume and number of streamlines generated, data from two control subjects were chosen at random. This analysis was performed in DSI Studio with the same tracking parameters that were used to generate the whole-brain networks. In order to capture a mixture of connection types, streamlines were generated and counted between the left inferior parietal lobe and the left superior parietal lobe (intra-hemispheric, U-shaped streamlines), the left entorhinal cortex and the left parahippocampal cortex (intra-hemispheric, non-U-shaped streamlines), and the right rostral anterior cingulate and the left frontal pole (inter-hemispheric streamlines). The shape of these connections were determined through visual inspection in DSI Studio. Although DTI datasets do not carry directional information, these tracts were generated in only one direction to examine whether seed ROI volume after FA thresholding is related to the number of streamlines generated between two ROIs. The results from this exploration are shown in Table 4.

**Figure 3:**
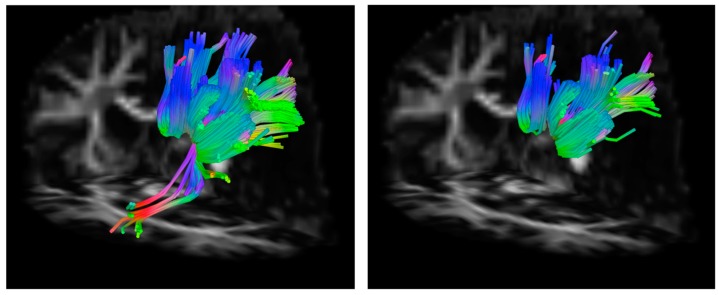
Example of streamlines generated from a WM seed versus a GM seed. (**left**) Streamlines generated from the WM left inferior parietal ROI. (**right**) Streamlines generated from the GM left inferior parietal ROI. The greater number of streamlines generated from the WM seed region is clear. Additionally, streamlines that connect part of the inferior parietal ROI to the superior parietal ROI that are not captured when seeding in the GM ROI are shown. GM: gray matter, ROI: region of interest, WM: white matter.

In subject 1, the difference in the number of streamlines generated between each pair of ROIs when seeding in WM ROIs compared to GM ROIs is much larger than the difference in the number of voxels between WM ROIs and GM ROIs after FA thresholding. Furthermore, a greater number of streamlines were generated between the left inferior parietal lobe and left superior parietal lobe when seeding in the left inferior parietal WM ROI, which contained a greater number of voxels after FA thresholding than the left inferior parietal GM ROI (see Table 4). Figure 3 shows a comparison of the tracts generated between the left inferior parietal lobe and left superior parietal lobe using GM and WM ROIs in subject 1. Qualitatively, the streamlines that form the tract between each of these ROI pairs traverse highly similar paths regardless of whether seeding was performed in the left inferior parietal WM or GM ROI. In subject 2, with the exception of the entorhinal cortex, fewer voxels in each WM ROI is associated with a greater number of streamlines generated between that WM ROI and the chosen target ROI. Similar to subject 1, the magnitude of the volume difference between WM and GM seed ROIs is smaller than the magnitude in the difference in the number of streamlines generated between the seed and target region. This suggests that seed ROI volume (determined in part by FA-thresholding) has an effect on the number of streamlines generated but that other factors are involved. The data from these two subjects demonstrate that there is a complex relationship between ROI shape, ROI location, individual anatomy, and the number of streamlines generated between two ROIs.

### 3.3. WM-Seed and GM-Seed Weighted Whole-Network Measures

Because the WM-seed and GM-seed networks showed significantly different densities and average strengths in within-group comparisons, we further examined whether global network measures in weighted networks differed between WM-seed and GM-seed networks within each group. Small-world properties of these networks were also determined. Table 5 shows the average global efficiency and average local efficiency for WM-seed and GM-seed networks and the equivalent random networks in both groups.

Global efficiency was significantly greater in WM-seed than GM-seed networks in both controls and individuals with AD (*p* = 3.26 × 10^−6^, *p* = 1.4 × 10^−7^). Local efficiency was also greater in WM-seed compared to GM-seed networks in both controls and individuals with AD (*p* = 2.23 × 10^−6^, *p* = 8.19 × 10^−6^). 

A small-world network is defined as having a global efficiency slightly smaller than that of a random network and a local efficiency greater than that of a random network [64,65]. As Table 5 shows, this is true, on average, for both WM-seed and GM-seed networks in both controls and AD. More importantly, the global and local efficiency measures of each subject’s WM-seed and GM-seed networks satisfied these small-world criteria, confirming that each network generated for every subject can be considered a small-world network at the individual level (see Table S3).

Between-group differences in whole-network efficiency measures using WM-seed or GM-seed networks were also examined. Both the global and the local efficiency were decreased in AD compared to controls using WM-seed networks (E_glob_: *p* = 0.0881, d = 0.664, E_loc_: *p* = 0.101, d = 0.6) and GM-seed networks (E_glob_: *p* = 0.317, d = 0.205, E_loc_: *p* = 0.604, d = 0.12). These comparisons did not reach significance, but the effect size was larger for the comparisons performed using WM-seed networks.

### 3.4. Between-Group Differences Using WM-Seed and GM-Seed Networks

**Figure 4:**
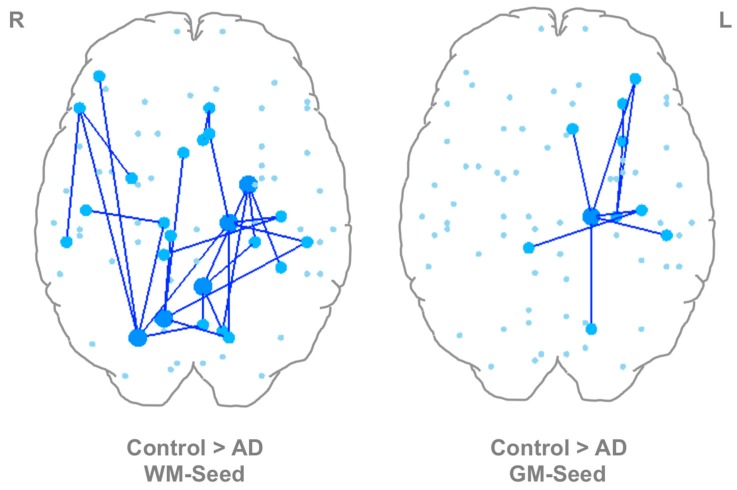
Between-Group Comparisons using WM-Seed and GM-Seed Networks. (**left**) Control > AD, WM ROIs. Nodes with a high number of differential connections are the left entorhinal cortex, left isthmus cingulate, left thalamus, right precuneus, and right superior parietal lobule. (**right**) Control > AD, GM ROIs. The left thalamus was the only node with a high number of differential connections. Large dark blue nodes have 4 or more differential connections, medium light blue nodes have 1–3 differential connections. Small light blue nodes were not part of the significant network. AD: Alzheimer’s disease, GM: gray matter, ROIs: regions of interest, WM: white matter.

The effect of seed location on network changes in AD compared to normal aging was determined. A network showing significant decreases in AD compared to controls was observed when comparisons were performed between both the WM-seed (*p* = 0.004) and GM-seed (*p* = 0.0326) networks. The network generated from the WM-seed network comparison contained 25 nodes and 27 edges (see Figure 4 and Table S4). The network generated from the GM-seed network comparison contained 10 nodes and 9 connections (see Figure 4 and Table S4). Five out of the nine connections in this network were present in the larger network generated by the WM-seed network comparison. This suggests that some unique features may be captured by the GM-seed networks despite the fact that a much larger and more significant network was found when the WM-seed networks were compared. No significant networks were found that were greater in AD relative to controls using either seeding method.

### 3.5. Targeted Node-Based Differences between Controls and AD

Lastly, the effect of seed location (e.g., GM or WM) on differences in nodal efficiency between AD and control groups was investigated. The targeted nodes were determined from the results presented in Section 3.4. Nodal efficiency was calculated for nodes with 4 or more and 2 or more differential connections in the WM-seed and GM-seed NBS networks, respectively, that showed decreases in AD compared to controls. The criteria for selecting nodes for this analysis differed for the NBS networks generated using WM-seed and GM-seed networks to ensure that the nodal efficiency of an equal number of nodes were tested from each network. The nodes included in this analysis are shown in Table 6. 

When using WM-seed networks, the nodal efficiency of the left entorhinal cortex (*p* = 0.0066), left thalamus (*p* = 0.041), left precentral gyrus (*p* = 0.0224), left rostral middle frontal cortex (*p* = 0.0065), and left hippocampus (*p* = 0.0121) were all significantly reduced in AD compared to controls (Table 7). The left entorhinal cortex (*p_adj_* = 0.0297), left rostral middle frontal cortex (*p_adj_* = 0.0297), and left hippocampus (*p_adj_* = 0.0363) survived false discovery rate (FDR) correction for multiple comparisons. The left precentral gyrus nearly survived FDR correction with a *p_adj_* equal to 0.0504.

When using GM-seed networks, the nodal efficiency of the left thalamus (*p* = 0.005) and left precentral gyrus (*p* = 0.0383), were significantly reduced in AD compared to controls. Only the left thalamus survived FDR correction with a *p_adj_* equal to 0.045. Similar to the power calculations presented for the global and local efficiency comparisons, the Cohen’s d values for the WM-seed network comparisons were stronger for all nodes except for the left thalamus (see Table S5).

## 4. Discussion

### 4.1. The Effects of Seed Location on Properties of WM-Seed and GM-Seed Networks

The results presented in Section 3.1 show that the WM-seed networks have a greater number of connections than the GM-seed networks in both AD and controls. Furthermore, most of the connections present in the GM-seed networks are also present in the WM-seed networks in both groups. Network density and network strength are higher in WM-seed than in GM-seed networks in both AD and controls. Network density is directly related to the number of connections present within a network and network strength is indirectly related to the number of connections within a network. Thus, it makes sense that both density and strength were higher in the WM-seed networks, which contained more connections.

The NBS results presented in Section 3.2 and Figure 2 complement the data in Section 3.1 and show that there is a significantly stronger subnetwork of regions (with some overlap) in WM-seed networks compared to GM-seed networks in controls and AD. Interestingly, the superior parietal cortex and insula were among the nodes with the highest number of differential connections in both the control and AD WM-seed networks. This suggests that for some regions, seeding in GM ROIs is unable to capture many of the connections between those regions and the rest of the brain. This could be due to a greater amount of partial voluming with CSF in these GM ROIs, large differences in the directions of the primary eigenvectors of the voxels in the GM ROIs compared to those of the voxels in the WM ROIs (which would result in streamlines failing the angle threshold), or differences in the three-dimensional geometry of these GM ROIs relative to their corresponding WM ROIs that significantly affect the number of streamlines generated from these regions. Further exploration of this in two control subjects (Table 4) suggested that FA-thresholded seed ROI volume does have an effect on the number of streamlines generated, but that there is a complex relationship between ROI shape, ROI location, individual anatomy, and the number of streamlines generated between two ROIs. These factors should be investigated in future research to better understand biases that may be present in between-group structural network comparisons.

Comparisons of global and local efficiency calculated from WM-seed and GM-seed networks followed a similar trend to that found with network strength and density in both controls and AD. Both global and local efficiency were stronger in WM-seed networks compared to GM-seed networks in both controls and AD. These results are in accordance with the results in Section 3.1, which show that the WM-seed networks have both a greater number of connections and, on average, stronger connections (i.e., a greater number of streamlines generated between regions) than the GM-seed networks. 

Overall, these results show that both WM-seed and GM-seed networks satisfy small-world criteria, but that there are systematic differences in the construction of WM-seed and GM-seed networks and the weighting within them in both AD and controls.

### 4.2. Differences between AD and Controls and the Effects of Seed Location on These Differences

Our main question in this study was how seed location affects between-group structural network comparisons. We examined this at both the network and nodal levels and found that, in most cases, comparisons between AD and controls using WM-seed networks had stronger effect sizes than comparisons using GM-seed networks.

Network strength and density were not significantly different between AD and controls using WM-seed networks. However, both measures were lower in AD compared to controls, which is the expected direction of difference. While network strength was not different between AD and controls using GM-seed networks, network density was lower in AD compared to controls using GM-seed networks and this approached significance (*p* = 0.051). This could be related to the effect of AD-related cortical thinning on the FA of GM seeds. Cortical thinning is apparent in individuals with AD compared to healthy aged individuals [69,70,71] and this was also true in our sample (AD_thickness_ = 2.13 mm, Control_thickness_ = 2.27 mm, *p* = 0.008). Because transformation from T1 to DTI space is based on the gray matter-white matter boundary, a thinner cortex would lower the FA of GM seeds due to partial voluming with CSF. In this case, network density could be artificially deflated in an AD population as a result of thinner cortex. This illustrates one aspect of the importance of parameter choice on between-group comparisons involving neurodegenerative disorders.

Global and local efficiency are two measures commonly used to characterize and compare weighted networks. We compared the global and local efficiency of controls and AD using WM-seed and GM-seed networks. The global and local efficiency were lower in AD relative to controls in all comparisons (except for the local efficiency of GM-seed networks), but none of these differences were significant. Though these p values are not significant, the p values corresponding to the WM-seed network between-group comparisons are lower than those corresponding to the GM-seed network comparisons. The effect size is also larger for the efficiency comparisons using WM-seed networks (E_glob_: *d* = 0.664, E_loc_: *d* = 0.6, medium effect size) versus GM-seed networks (E_glob_: *d* = 0.205, E_loc_: *d* = 0.12, small effect size). To have an 80% chance of finding a significant difference between these measures using DTI data and this set of network construction parameters would require approximately 33-45 subjects if WM-seed networks were used. In contrast, over 390 subjects would be needed if GM-seed networks were used. This suggests WM-seed networks might be more sensitive to differences between AD and controls when comparing whole-network measures. This could be due to the greater number of streamlines between regions captured by WM-seed networks. With a greater sample size, it is expected that the trends in our results would reach significance as lower network efficiency in weighted structural networks in individuals with AD compared to controls has been reported in the literature [37,42]. 

Seed location affected the NBS results of the comparisons between controls and AD. The network generated from the WM-seed network comparison was larger and more significant than that generated from the GM-seed network comparison. The WM-seed network comparison result included nodes that are known to be affected in AD such as the entorhinal cortex [23,72,73], isthmus of the cingulate [74,75,76], precuneus [21,24,77], and thalamus [78,79,80]. The network generated from the GM-seed network comparison was largely localized to the left hemisphere and also included nodes known to be affected in AD, such as the hippocampus [72,81], thalamus, and temporal pole [82,83]. This network was smaller and less significant than that found when using the WM seeds. Despite this, four out of the nine connections in this network were present in the larger network generated by the WM-seed network comparison, suggesting that some unique features may be captured by the GM-seed networks. This is also reflected in the small number of connections (~45) that were found to be unique to the GM-seed networks in each subject (see Table 1).

Interestingly, the left thalamus showed five differential connections in both significant networks and four out of these five connections were the same in both seeding conditions. The GM-seed network contained a connection between the left thalamus and the left rostral middle frontal cortex, which was not present in the WM-seed network. Conversely, the WM-seed network contained a connection between the left thalamus and the right superior parietal cortex that was not present in the GM-seed network. Because the thalamus is a subcortical ROI, the same ROI was used in generating both the WM-seed and GM-seed networks. Thus, it makes sense that similar differences between controls and AD would be present with respect to this node. However, even though the hippocampus ROI was also the same in both WM and GM seeding conditions, it was present in the network generated when GM-seed networks were compared but not when WM-seed networks were compared. This additionally reflects the possibility that GM-seed networks capture some unique and important features that the WM-seed networks do not.

Wang et al. [42] recently used DTI and high angular resolution diffusion imaging (HARDI) [84] to assess differences in structural networks between controls and individuals with AD. They found weaker connections associated with the right thalamus, left hippocampus, bilateral precuneus, and right parahippocampal gyrus, among other regions. The NBS networks shown in Figure 4 similarly include the bilateral thalamus, left hippocampus, bilateral precuneus, and left hippocampal gyrus. Despite methodological differences (e.g., diffusion encoding scheme, fiber orientation reconstruction method, and statistical method), connections to similar regions were found in the present study and Wang et al. [42].

Lastly, the effect of seed location on the nodal efficiency of 9 nodes was examined. These nodes were selected based on the NBS results of the comparisons between controls and AD. Similar to the global and local efficiency results, the effect sizes of 9 out of 10 comparisons of nodal efficiency between controls and AD were larger for comparisons that used the WM-seed networks. The only comparison with an effect size larger for GM-seed networks was the thalamus. Regardless, the nodal efficiency of the thalamus was found to be lower in AD compared to controls using both WM-seed and GM-seed networks, which is the expected result.

Lo et al. [37] examined differences in the nodal efficiency of structural networks built from DTI data between controls and individuals with AD. They found significantly reduced nodal efficiency of nodes primarily in the right frontal lobe and in the right temporal pole. We found significantly reduced nodal efficiency in a left frontal lobe and the decreased nodal efficiency in the left temporal pole in AD approached significance. Lo and colleagues also found significantly reduced nodal efficiency in the left superior frontal gyrus, which in addition to other frontal regions was included in our NBS networks. In contrast to Lo et al., our nodal efficiency assessments were targeted by our NBS results, thus limiting the number of statistical comparisons and the nodes tested. Furthermore, our network weights were derived from the number of streamlines between regions while Lo et al.’s networks were weighted by both number of streamlines and FA – fundamentally changing what the connections represent [14]. Wang et al. [42] also examined differences in nodal efficiency between controls and AD and found reduced nodal efficiency in the left temporal pole, among other regions. We found decreased nodal efficiency in the left temporal pole in AD relative to controls that approached significance. Similar to Lo et al., more overlap in decreased nodal efficiency may have been found if the methodologies used between our study and Wang et al. were more similar.

A greater number of the nodes examined—including the entorhinal cortex and hippocampus—had significantly reduced nodal efficiency in AD compared to controls when using WM-seed networks as opposed to when using GM-seed networks. Furthermore, the observed reductions were more significant when utilizing WM-seed compared to GM-seed networks to perform the comparisons. These results are in agreement with the global efficiency, local efficiency, and NBS results. Together the results indicate that WM-seed networks produce a greater number of and more highly significant differences in between-group comparisons of controls and AD.

## 5. Conclusions

The data presented demonstrate that seed location (i.e., GM or WM) has an effect on both network construction and subsequent between-group network analyses. When forming whole-brain structural networks using a seed-to-target ROI-based strategy and deterministic tractography, seeding in WM ROIs produces stronger networks with a greater number of connections than seeding in GM ROIs. In the present study, freely-available and widely used GM and WM parcellations within FreeSurfer were used to construct network nodes. However, this is not necessarily the ideal parcellation for structural connectivity analyses or studies of aging and AD. Likely, the choice of parcellation will reflect the goals of a particular study and parcellation schemes for use in connectivity studies are continuing to evolve [85]. Furthermore, it has been shown that the number of nodes affects network properties and graph theory measures more than the specific parcellation scheme (see [14] for review). Thus, our results suggest that seeding in white matter regions adjacent to gray matter regions, though they may be divided according to a different parcellation scheme, is nevertheless preferable.

Between-group comparisons of whole-brain networks, whole-network measures, and node-based measures produced a greater number of and more strongly significant differences between AD and control groups when WM-seed networks were used. WM-seed networks had a greater number of connections than GM-seed networks in both AD and controls and the vast majority of connections in GM-seed networks were present in WM-seed networks. A small number of connections were present in GM-seed networks that were not present in WM-seed networks. Though the GM-seed between-group NBS result was less significant and less extensive compared to the WM-seed NBS result, it did include nodes such as the hippocampus and temporal pole (not present in the WM-seed NBS result) that are affected in AD. This suggests that it may be beneficial to include the ~50 unique connections captured in each subject’s GM-seed network in his/her WM-seed network to more fully capture the underlying structural network. While this particular strategy has not been investigated or suggested in the literature, the idea of producing multiple networks for each subject in order to isolate or capture important features has been suggested and used [12,14]. Our strategy is one example that may be useful in structural network studies and should be systematically investigated in future work.

Overall, our results suggest that seeding in WM ROIs captures more of the underlying streamlines between regions than seeding in GM ROIs and highlights the importance of parameter choice on between-group structural network comparisons. It is recognized that parameter and methodology selection in structural network studies is a hard problem with a number of solutions, some of which may be more appropriate for a given application than others [10,12,85]. Thus, papers that have assessed the effect of parameter choice on network properties, including this study, have tended to focus on the effects of one parameter or methodology on network properties [8,9,10]. A better understanding of the effects of parameter choice on networks constructed using different methodologies will provide investigators with the tools necessary to make more informed decisions on parameters in order to maximize the power of their experiments. If we are to use these highly informative network-based methods moving forward in the study of disease, evaluation of therapeutic interventions, or as a diagnostic tool, the methodology used to construct whole-brain structural networks must be in the forefront. It is unreasonable to expect that the field will adopt one method as a network-construction standard. Despite this, further study into the effects of various network-construction parameters on between-group comparisons are warranted to better understand how parameters affect the ability of networks and network measures to detect differences.

## Figures and Tables

**Table 1:** Number of Connections for WM-Seed and GM-Seed Networks and the Percentage of GM-Seed Connections in WM-Seed Networks.

	WM-Seed Total Connections	GM-Seed Total Connections	WM-Seed Connections Not in GM-Seed	GM-Seed Connections Not in WM-Seed	Percent of GM-Seed Connections in WM-Seed Networks
Control	1110 (80)	776 (49)	380 (59)	46 (21)	94.1% (2.5%)
AD	1089 (74)	723 (64)	411 (51)	45 (17)	93.7% (2.5%)

Values listed correspond to mean (standard deviation). AD: Alzheimer’s disease, GM: gray matter, WM: white matter.

**Table 2:** Average Network Density and Strength.

	WM-Seed Network Density	GM-Seed Network Density	WM-Seed Average Network Strength	GM-Seed Average Network Strength
Control	0.195 (0.0141) *	0.136 (0.00858)	1361.5 (307.9) *	572.4 (117.6)
AD	0.191 (0.0131) *	0.127 (0.0112)	1251.6 (197.3) *	579.9 (99.2)

Values listed correspond to mean (standard deviation). * *p* < 10^−6^ within-group comparisons between WM-seed and GM-seed networks. AD: Alzheimer’s disease, GM: gray matter, WM: white matter.

**Table 3:** Nodes with 6 or More Differential Connections in WM-Seed > GM-Seed NBS Results in Controls and AD.

Node	Number of Differential Connections
Controls	
Left precentral	6
Left superior parietal	7
Left insula	8
Right inferior parietal	6
Right lateral occipital	6
Right superior parietal	7
Right insula	11
AD	
Left superior parietal	7
Right superior parietal	7
Right insula	6

AD: Alzheimer’s disease, GM: gray matter, NBS: Network Based Statistic, WM: white matter.

**Table 4:** Relationship between FA-Thresholded Seed ROI Volume and Number of Streamlines Generated between ROIs.

Seed ROI	FA-Thresholded Seed ROI Volume (Voxel Number)	Number of Streamlines Generated between Seed ROI and GM Target ROI
Subject 1		
WM left inferior parietal lobe	304	904
GM left inferior parietal lobe	269	264
WM left entorhinal cortex	20	1248
GM left entorhinal cortex	48	45
WM right rostral anterior cingulate	55	112
GM right rostral anterior cingulate	68	9
Subject 2		
WM left inferior parietal lobe	275	99
GM left inferior parietal lobe	276	66
WM left entorhinal cortex	22	232
GM left entorhinal cortex	32	456
WM right rostral anterior cingulate	63	61
GM right rostral anterior cingulate	82	6

FA: fractional anisotropy, GM: gray matter, ROI: region of interest, WM: white matter.

**Table 5:** Weighted Global Network Measures.

Network	Weighted Global Efficiency	Weighted Global Efficiency (Random)	Weighted Local Efficiency	Weighted Local Efficiency (Random)
Control WM-seed	72.8 (20.3)	84.7 (20.3)	52.8 (11.3)	30.6 (7.78)
Control GM-seed	29.2 (7.47)	36.5 (7.89)	26.2 (4.52)	11.5 (3.01)
AD WM-seed	62.6 (10.4)	77.5 (12.6)	46.8 (8.71)	27.3 (4.71)
AD GM-seed	27.9 (5.23)	36.5 (6.54)	26.8 (5.38)	10.5 (2.36)

Values listed correspond to mean (standard deviation). AD: Alzheimer’s disease, GM: gray matter, WM: white matter.

**Table 6:** Nodes Used in Nodal Efficiency Comparisons.

WM-Seed Network Node	Degree in Significant NBS Network
Left entorhinal cortex	4
Left isthmus cingulate	5
Left thalamus	5
Right precuneus	4
Right superior parietal cortex	5
GM-Seed Network Node	Degree in Significant NBS Network
Left precentral gyrus	2
Left rostral middle frontal gyrus	2
Left temporal pole	2
Left hippocampus	2
Left thalamus	5

GM: gray matter, NBS: Network Based Statistic, WM: white matter.

**Table 7:** Average Nodal Efficiency WM-Seed and GM-Seed Networks.

Node	Control WM-Seed Nodal Efficiency	AD WM-Seed Nodal Efficiency	Control GM-Seed Nodal Efficiency	AD GM-Seed Nodal Efficiency
Left entorhinal	108 (44.3) **	63.2 (27.5)	51.2 (26.5)	35.2 (14.8)
Left isthmus cingulate	81.6 (32.5) *	62.3 (16.1)	28.8 (10.5)	29.5 (9.44)
Left thalamus	89.9 (33.4) *	66.1 (23.1)	52.2 (20.3) **	31.1 (11.1)
Right precuneus	97.1 (41)	74.1 (29.6)	28.4 (11.5)	25.9 (9.15)
Right superior parietal cortex	103 (35.7)	88.6 (30.4)	32.2 (10.4)	31.8 (8.8)
Left precentral gyrus	106 (30.4) *	80.9 (20.3)	49.4 (17.5)	37.1 (11)
Left rostral middle frontal gyrus	66.7 (14.4) **	51.7 (9.46)	28.2 (5.44)	25.6 (7.48)
Left temporal pole	95.6 (35) *	73.6 (25.1)	52.9 (16.3)	44.1 (14.2)
Left hippocampus	95 (25.5) **	67.1 (25.2)	40.2 (14.4)	31.3 (13.4)

Values listed correspond to mean (standard deviation). * FDR adjusted *p* value < 0.08 for Control > AD, WM-seed or GM-seed comparisons ** FDR adjusted *p* value < 0.05 for Control > AD, WM-seed or GM-seed comparisons. AD: Alzheimer’s disease, FDR: false discovery rate, GM: gray matter, WM: white matter.

## Supplementary Materials

The following are available online at www.mdpi.com/2076-3425/7/4/37/s1, Table S1: ADNI Database Subject Numbers, Ages, and Global Clinical Dementia Rating (CDR) Scale Score, Table S2: Full List of Connections in Control WM > GM and AD WM > GM Significant NBS Networks, Table S3: E_glob_ and E_loc_ values from each subject’s WM-seed and GM-seed Networks, Table S4: Full List of Connections in Control > AD WM-Seed and Control > AD GM-Seed Significant NBS Networks, Table S5: Uncorrected p Values and Cohen’s d Values from Control > AD WM-Seed and GM-Seed Targeted Nodal Efficiency Comparisons.
