# Supplementary material for: Seed Location Impacts Whole-Brain Structural Network Comparisons between Healthy Elderly and Individuals with Alzheimer’s Disease
Source: Brain Sci. 2017 Apr 6;7(4):37. doi: 10.3390/brainsci7040037 (PMC5406694; doi:10.3390/brainsci7040037)
Supplement: Supplementary file 1 [file brainsci-07-00037-s001.docx]

**Supplementary Materials**

**Table S1.** Alzheimer’s Disease Neuroimaging Initiative (ADNI) Database subject numbers, ages and global Clinical Dementia Rating (CDR) scale score.

| **Subject** | **Dx** | **Age** | **CDR** |
| --- | --- | --- | --- |
| 003_S_4119 | control | 79.4 | 0 |
| 003_S_4350 | control | 73 | 0 |
| 003_S_4441 | control | 68.8 | 0 |
| 003_S_4839 | control | 65.6 | 0 |
| 003_S_4555 | control | 65.9 | 0 |
| 003_S_4644 | control | 67.7 | 0 |
| 003_S_4840 | control | 61.8 | 0 |
| 007_S_4488 | control | 72.7 | 0 |
| 007_S_4516 | control | 71.4 | 0 |
| 003_S_4872 | control | 68.6 | 0 |
| 003_S_4152 | AD | 60.8 | 2 |
| 016_S_4353 | AD | 55.7 | 1 |
| 003_S_4136 | AD | 66.8 | 1 |
| 003_S_4373 | AD | 71.2 | 2 |
| 003_S_5165 | AD | 78.9 | 1 |
| 003_S_5187 | AD | 61.5 | 1 |
| 007_S_4911 | AD | 74.6 | 1 |
| 016_S_4009 | AD | 90.4 | 1 |
| 003_S_4892 | AD | 75.2 | 1 |
| 016_S_4591 | AD | 66 | 1 |

**Table S2.** Full list of connections in control WM > GM and AD WM > GM significant NBS networks.

| **Controls WM > GM**  **Node A** | **Node B** |
| --- | --- |
| lh banks sts* | lh middle temporal |
| lh banks sts * | lh superior temporal |
| lh banks sts | lh supramarginal |
| lh caudal middle frontal | lh pars opercularis |
| lh caudal middle frontal * | lh precentral |
| lh caudal middle frontal | lh rostral middle frontal |
| lh cuneus | lh pericalcarine |
| lh cuneus * | lh superior parietal |
| lh entorhinal | lh parahippocampal |
| lh entorhinal | lh temporal pole |
| lh fusiform * | lh lateral occipital |
| lh inferior parietal | lh inferior temporal |
| lh inferior parietal * | lh lateral occipital |
| lh inferior parietal * | lh middle temporal |
| lh inferior parietal * | lh superior parietal |
| lh inferior parietal * | lh supramarginal |
| lh inferior temporal * | lh lateral occipital |
| lh inferior temporal | lh middle temporal |
| lh isthmus cingulate | lh precuneus |
| lh isthmus cingulate | lh hippocampus |
| lh lateral occipital | lh pericalcarine |
| lh lateral occipital | lh superior temporal |
| lh lateral orbitofrontal | lh medial orbitofrontal |
| lh lateral orbitofrontal | lh insula |
| lh middle temporal | lh superior parietal |
| lh parahippocampal | lh temporal pole |
| lh parahippocampal | lh hippocampus |
| lh paracentral | lh postcentral |
| lh paracentral | lh posterior cingulate |
| lh paracentral | lh precentral |
| lh pars opercularis | lh precentral |
| lh pars opercularis | lh superior frontal |
| lh pars triangularis | lh superior parietal |
| lh pars triangularis | lh insula |
| lh postcentral * | lh precentral |
| lh postcentral * | lh superior parietal |
| lh postcentral * | lh supramarginal |
| lh postcentral | lh insula |
| lh precentral * | lh superior frontal |
| lh precentral | lh insula |
| lh precuneus | lh superior frontal |
| lh precuneus * | lh superior parietal |
| lh rostral middle frontal * | lh superior frontal |
| lh rostral middle frontal | lh insula |
| lh rostral middle frontal | rh superior frontal |
| lh superior frontal | rh caudal anterior cingulate |
| lh superior parietal * | rh precuneus |
| lh superior temporal | lh supramarginal |
| lh superior temporal * | lh insula |
| lh insula * | lh putamen |
| lh insula * | lh thalamus proper |
| rh banks sts * | rh middle temporal |
| rh cuneus | rh pericalcarine |
| rh fusiform | rh inferior temporal |
| rh fusiform | rh lateral occipital |
| rh inferior parietal | rh inferior temporal |
| rh inferior parietal | rh lateral occipital |
| rh inferior parietal * | rh middle temporal |
| rh inferior parietal * | rh superior parietal |
| rh inferior parietal * | rh supramarginal |
| rh inferior temporal | rh lateral occipital |
| rh isthmus cingulate | rh precuneus |
| rh isthmus cingulate * | rh hippocampus |
| rh lateral occipital | rh lingual |
| rh lateral occipital | rh middle temporal |
| rh lateral occipital * | rh superior parietal |
| rh lingual | rh middle temporal |
| rh lingual | rh pericalcarine |
| rh lingual | rh hippocampus |
| rh middle temporal * | rh superior temporal |
| rh parahippocampal * | rh hippocampus |
| rh paracentral | rh postcentral |
| rh paracentral * | rh precentral |
| rh paracentral | rh superior frontal |
| rh pars opercularis | rh superior frontal |
| rh pars opercularis | rh insula |
| rh pars triangularis | rh superior parietal |
| rh pars triangularis | rh insula |
| rh postcentral * | rh precentral |
| rh postcentral | rh supramarginal |
| rh postcentral | rh insula |
| rh precentral * | rh superior frontal |
| rh precentral * | rh insula |
| rh precuneus * | rh superior parietal |
| rh precuneus | rh superior temporal |
| rh rostral middle frontal | rh insula |
| rh superior parietal * | rh superior temporal |
| rh superior parietal | rh supramarginal |
| rh superior parietal * | rh insula |
| rh superior temporal * | rh supramarginal |
| rh superior temporal * | rh insula |
| rh supramarginal | rh insula |
| rh temporal pole | rh insula |
| rh insula * | rh putamen |
| rh insula | rh thalamus proper |
| **AD WM > GM** |  |
| lh banks sts * | lh middle temporal |
| lh banks sts * | lh superior temporal |
| lh caudal middle frontal * | lh precentral |
| lh caudal middle frontal | lh superior frontal |
| lh cuneus * | lh superior parietal |
| lh fusiform * | lh lateral occipital |
| lh inferior parietal * | lh lateral occipital |
| lh inferior parietal * | lh middle temporal |
| lh inferior parietal * | lh superior parietal |
| lh inferior parietal * | lh supramarginal |
| lh inferior temporal * | lh lateral occipital |
| lh postcentral * | lh precentral |
| lh postcentral * | lh superior parietal |
| lh postcentral * | lh supramarginal |
| lh precentral * | lh superior frontal |
| lh precentral | lh supramarginal |
| lh precuneus * | lh superior parietal |
| lh precuneus | lh insula |
| lh rostral middle frontal * | lh superior frontal |
| lh rostral middle frontal | lh superior parietal |
| lh superior frontal | lh insula |
| lh superior parietal | lh supramarginal |
| lh superior parietal * | rh precuneus |
| lh superior temporal * | lh insula |
| lh insula * | lh putamen |
| lh insula * | lh thalamus proper |
| rh banks sts * | rh middle temporal |
| rh banks sts | rh superior temporal |
| rh caudal middle frontal | rh pars opercularis |
| rh caudal middle frontal | rh superior frontal |
| rh inferior parietal * | rh middle temporal |
| rh inferior parietal * | rh superior parietal |
| rh inferior parietal * | rh supramarginal |
| rh inferior temporal | rh middle temporal |
| rh isthmus cingulate * | rh hippocampus |
| rh lateral occipital * | rh superior parietal |
| rh middle temporal | rh superior parietal |
| rh middle temporal * | rh superior temporal |
| rh parahippocampal * | rh hippocampus |
| rh paracentral * | rh precentral |
| rh pars opercularis | rh precentral |
| rh pars opercularis | rh superior frontal |
| rh pars orbitalis | rh pars triangularis |
| rh pars orbitalis | rh insula |
| rh pars triangularis | rh superior frontal |
| rh postcentral * | rh precentral |
| rh postcentral | rh superior parietal |
| rh precentral * | rh superior frontal |
| rh precentral * | rh insula |
| rh precuneus * | rh superior parietal |
| rh precuneus | rh insula |
| rh precuneus | rh hippocampus |
| rh superior parietal * | rh superior temporal |
| rh superior parietal * | rh insula |
| rh superior temporal * | rh supramarginal |
| rh superior temporal * | rh insula |
| rh insula * | rh putamen |

* Connections that are found in both networks, lh: left hemisphere, rh: right hemisphere, sts: superior temporal sulcus

**Table S3.** E_glob_ and E_loc_ values from each subject’s WM-seed and GM-seed networks.

| **Subject** | **E_glob_ WM-Seed** | **E_glob_ WM-Seed, (Mean 100 Random Networks)** | **Mean E_loc_, WM-Seed** | **Mean E_loc_, WM-Seed (Mean 100 Random Networks)** | **E_glob_ GM-Seed** | **E_glob_ GM-Seed, (Mean 100 Random Networks)** | **Mean E_loc_, GM-Seed** | **Mean E_loc_, GM-Seed (Mean 100 Random Networks)** |
| --- | --- | --- | --- | --- | --- | --- | --- | --- |
| 1 | 55.2941 | 70.3056 | 44.6921 | 24.9302 | 22.125 | 30.0353 | 22.3817 | 8.681 |
| 2 | 58.6357 | 71.0251 | 47.7294 | 25.2785 | 24.1178 | 33.7659 | 28.6879 | 9.7336 |
| 3 | 71.0093 | 83.0188 | 52.8003 | 30.101 | 29.2013 | 38.1594 | 27.7109 | 11.8098 |
| 4 | 121.5073 | 130.8333 | 81.7149 | 48.9809 | 43.5716 | 51.5419 | 35.8422 | 17.8143 |
| 5 | 84.031 | 104.1937 | 52.5258 | 34.1265 | 37.9799 | 47.0396 | 27.7768 | 14.4728 |
| 6 | 72.3692 | 79.3239 | 50.2713 | 29.7063 | 27.7967 | 33.8098 | 24.2227 | 11.711 |
| 7 | 73.7285 | 87.7555 | 55.6651 | 32.5498 | 27.5737 | 31.663 | 23.8261 | 10.1225 |
| 8 | 59.2431 | 70.1058 | 46.8837 | 25.3652 | 21.0352 | 25.8705 | 20.1887 | 9.413 |
| 9 | 51.3617 | 61.4333 | 39.897 | 21.0903 | 23.5322 | 33.0193 | 22.5937 | 7.8748 |
| 10 | 80.3244 | 88.779 | 55.5194 | 33.86 | 35.5072 | 40.4668 | 28.8272 | 13.1217 |
| 11 | 63.9122 | 77.6117 | 49.6722 | 29.5648 | 30.9963 | 40.4219 | 34.6991 | 13.9279 |
| 12 | 63.0731 | 95.0434 | 50.519 | 30.0529 | 32.2409 | 45.7703 | 33.9447 | 11.6876 |
| 13 | 61.064 | 71.0228 | 37.6417 | 25.2083 | 28.6639 | 35.1515 | 25.124 | 12.2314 |
| 14 | 65.1799 | 69.6933 | 40.652 | 26.5396 | 23.1266 | 31.7681 | 20.9168 | 9.6121 |
| 15 | 47.0359 | 67.6203 | 41.0301 | 21.4952 | 20.3741 | 28.9591 | 23.0693 | 6.606 |
| 16 | 68.08 | 91.4065 | 55.8083 | 29.8547 | 36.0644 | 47.8572 | 33.746 | 12.628 |
| 17 | 45.4608 | 56.5835 | 32.0537 | 18.0699 | 20.2227 | 29.0859 | 21.0446 | 7.2022 |
| 18 | 69.4134 | 81.2816 | 49.5432 | 30.6129 | 28.6006 | 34.5817 | 25.4826 | 10.0789 |
| 19 | 81.0507 | 93.2082 | 60.3773 | 34.0459 | 31.143 | 37.9692 | 26.7178 | 11.812 |
| 20 | 61.771 | 71.4198 | 50.6308 | 27.1647 | 27.0563 | 32.9537 | 23.272 | 9.6197 |

Each subject’s WM-seed and GM-seed networks satisfy small-world criteria with E_glob_ less than that of random networks and E_loc_ greater than that of random networks.

**Table S4.** Full list of connections in control > AD WM-seed and control > AD GM-seed significant
NBS networks.

| **Controls > AD, WM-Seed** |  |
| --- | --- |
| **Node A** | **Node B** |
| lh caudal anterior cingulate | lh rostral anterior cingulate |
| lh entorhinal | lh fusiform |
| lh entorhinal | lh isthmus cingulate |
| lh entorhinal | lh lingual |
| lh entorhinal | lh parahippocampal |
| lh isthmus cingulate | lh lingual |
| lh isthmus cingulate | lh parahippocampal |
| lh isthmus cingulate | lh precuneus |
| lh postcentral * | lh thalamus proper |
| lh postcentral | rh precuneus |
| lh precentral * | lh thalamus proper |
| lh precentral * | rh paracentral |
| lh precuneus | rh superior parietal |
| lh rostral anterior cingulate | lh superior frontal |
| lh superior frontal * | lh thalamus proper |
| lh superior frontal | rh caudal anterior cingulate |
| lh superior parietal * | lh thalamus proper |
| lh superior parietal | rh precuneus |
| lh thalamus proper | rh superior parietal |
| rh pars orbitalis | rh superior parietal |
| rh pars triangularis | rh postcentral |
| rh pars triangularis | rh superior parietal |
| rh pars triangularis | rh putamen |
| rh posterior cingulate | rh precuneus |
| rh precentral | rh thalamus proper |
| rh precuneus | rh rostral anterior cingulate |
| rh superior parietal | rh thalamus proper |
| **Controls > AD, GM-Seed** |  |
| lh lateral orbitofrontal | lh temporal pole |
| lh postcentral * | lh thalamus proper |
| lh precentral * | lh thalamus proper |
| lh precentral * | rh paracentral |
| lh rostral middle frontal | lh hippocampus |
| lh rostral middle frontal | lh thalamus proper |
| lh superior frontal * | lh thalamus proper |
| lh superior parietal * | lh thalamus proper |
| lh temporal pole | lh hippocampus |
| lh lateral orbitofrontal | lh temporal pole |

* Connections that are found in both networks

**Table S5.** Uncorrected *p*-values and Cohen’s d-values from control > AD WM-seed and GM-seed targeted nodal efficiency comparisons.

| **Node** | **Comparisons Using WM-Seed Networks (Uncorrected *p*, Control > AD)** | **Cohen’s d  (WM-Seed Networks,  Control > AD)** | | **Comparisons Using GM-Seed Networks (Uncorrected *p*, Control > AD)** | **Cohen’s d  (GM-Seed Networks,  Control > AD)** | |
| --- | --- | --- | --- | --- | --- | --- |
| Left entorhinal | 0.0066 | 1.25 | 0.0562 | | | 0.77 |
| Left isthmus cingulate | 0.0583 | 0.79 | 0.5618 | | | 0.07 |
| Left thalamus | 0.0405 | 0.84 | 0.005 | | | 1.34 |
| Right precuneus | 0.0839 | 0.65 | 0.2976 | | | 0.24 |
| Right superior parietal | 0.176 | 0.44 | 0.4651 | | | 0.042 |
| Left precentral | 0.0224 | 0.99 | 0.0383 | | | 0.86 |
| Left rostral middle frontal | 0.0065 | 1.26 | 0.1959 | | | 0.4 |
| Left temporal pole | 0.0614 | 0.73 | 0.1073 | | | 0.58 |
| Left hippocampus | 0.0121 | 1.1 | 0.0851 | | | 0.64 |

Effect sizes are larger in comparisons using WM-seed networks except for the left thalamus.
